# Introduction of the Charité Mobility Index (CHARMI) – A Novel Clinical Mobility Assessment for Acute Care Rehabilitation

**DOI:** 10.1371/journal.pone.0169010

**Published:** 2016-12-22

**Authors:** Max E. Liebl, Nancy Elmer, Isabelle Schroeder, Christine Schwedtke, Angelika Baack, Anett Reisshauer

**Affiliations:** 1 Department of Physical Medicine and Rehabilitation, Charité University Hospital, Berlin, Germany; 2 Charité Physiotherapy and Prevention Centre, Charité University Hospital, Berlin, Germany; Yokohama City University, JAPAN

## Abstract

**Introduction:**

Mobility is an essential part of a person’s functioning and independence. It encompasses locomotive functions, but also the more basic functions of positioning and transferring. Despite the availability of several mobility-related assessment instruments to date, there is a need for assessment instruments with the specific capability to display the full range of mobilisation. Our aim was to develop and validate a scoring instrument with hierarchical composition where every score value stands for a defined mobility level.

**Participants and Methods:**

A previously developed and validated pilot instrument was applied to assess patients (*n* = 113) admitted to an acute rehabilitation programme. Mobility was assessed during admission, subsequently at weekly intervals and at discharge to acquire a detailed status of mobility at multiple time points and individual mobilisation profiles over time. The scoring instrument was then remodelled based on clinical criteria to establish an easy-to-use scoring system with hierarchical composition. Psychometric properties were calculated using an independent sample of 87 consecutive patients.

**Results:**

Content validity could be affirmed. The psychometric tests demonstrated excellent convergent validity with the three mobility items of the Barthel Index (*r* = 0.93), despite an adequately lower correlation with the whole Barthel Index (*r* = 0.63). Adequate floor and ceiling effects (20%) and a large responsiveness to change (ǀ*d*ǀ = 1.7, *p* < 0.001) between admission and discharge values were demonstrated. Inter-rater reliability was excellent (*κ* = 0.88).

**Conclusions:**

The Charité Mobility Index (CHARMI) is a promising, easy-to-use hierarchical scoring instrument assessing the full individual spectrum from immobility to unlimited mobility, including positioning, transfer and locomotion items. It allows for monitoring of mobilisation.

## Introduction

Mobility in its classical meaning is the ability to move freely. It is essential for social activities and participation. From a clinical perspective, mobility encompasses locomotion abilities and a range of basic activities, such as changing and maintaining body positions, as displayed in the International Classification of Functioning, Disability and Health (ICF) [1].

In contrast, immobility is the cause and consequence of numerous medical complications [2,3]. It leads to an increase in morbidity and mortality by infectious diseases, pressure ulcers, contractures, thromboembolism, muscle atrophy, cardiopulmonary deconditioning, osteoporosis, cartilage degeneration and many other negative sequelae, affecting nearly every organ system or body structure [2–9]. Therefore, early mobilisation and rehabilitation should be an integral part of every medical treatment concept, trying to maintain or improve a patient’s functioning [10]. Early mobilisation and rehabilitation are associated with an improved functional status and a shorter hospital stay [11–15]. However, this correlation is not limited to the acute phase. An increase in gait speed by training is predictive for a substantial reduction of mortality in elderly patients [16].

Between complete immobility and full mobility, there is a broad spectrum of individual locomotive competencies, ranging from positioning in the bed, transfer to an upright position and unimpaired movement. An analysis of the existing mobility assessment instruments reveals the lack of a simple and practical but reliable assessment tool displaying the whole range of mobility. There is a variety of existing tools, measuring different operationalised aspects of mobility or mobility-associated competencies. Many of them regard mobility as a part of a broader test battery; or they are originally designed for specific diseases (e.g. Rivermead Motor Assessment, Barthel Index, Functional Independence Measure) [17–22]. Some assessment instruments test single mobility-related tasks as an indicator of global mobility (e.g. Timed Up and Go Test) and therefore have high floor and ceiling effects, as well as a limited scale width in the acute care setting [23–25]. Some focus on other mobility aspects, such as safety (e.g. Tinetti Test, Berg Balance Scale), requirement of assistance (e.g. Functional Ambulation Categories), or restricted high-level motoric functions (e.g. High Level Mobility Assessment Tool HiMAT) [26–31]. Only a few display the full spectrum of mobility, such as the Elderly Mobility Scale (EMS), designed and validated for geriatric patients, and the de Morton Mobility Index (DEMMI) [32–35]. The latter allows for a measurement using a Rasch analysis-calculated (pseudo-)interval scale, which is an attractive feature, especially for scientific use. However, the score requires a controlled assessment setting and is therefore not very practical in clinical use. In addition, both the DEMMI and the EMS do not consider relevant mobility tasks, such as climbing stairs and wheelchair mobility.

Another widely used global mobility assessment is the Rivermead Mobility Index (RMI) [36]. It is an extension of the Rivermead Motor Assessment and poses 15 mobility-related questions, in which each positively answered questions gains one point. The sum score is used as a measure of mobility. The RMI encompasses a wide range of the mobility spectrum and is based on the patients’ functional independence. A general problem regarding the use of sum-scores is that the achieved score alone does not allow for reliable interpretation of the mobility level, except for the highest and lowest sum-scores. For example, a sum score of 7 in the RMI may be interpreted as limited mobility, but it does not describe which functions precisely are impaired.

Therefore, our aim was to develop and validate a novel, easy-to-use assessment instrument based on ICF categories, which displays the whole spectrum of mobility in therapy-relevant increments of function, and includes a hierarchical composition of the items scored.

## Materials and Methods

### Pilot Phase

In a first study phase, a pilot instrument was developed and validated in a small sample (*n* = 36, previously published) [37]. This pilot instrument had seven independent (i.e. non-hierarchical) mobility items, each with an intra-item rating in the form of a Likert scale. The items were collected from existing assessment instruments. They were *transfers in bed*, *sitting on edge of bed*, *transfer from bed to chair*, *wheelchair mobility*, *standing*, *walking* and *climbing stairs*. The score was applied as a standard assessment tool within the acute care rehabilitation (ACR) programme of a university hospital. Over the course of 1 year 113 immobile patients were consecutively assessed during admission, at weekly intervals and at discharge. Every mobility task was assessed according to the time and resources needed for mobilisation, such as assistance or mobilisation aids. The resultant longitudinal mobilisation profiles were documented. Based on these profiles, our novel hierarchical assessment tool was developed.

### Development Phase

Table 1 displays the criteria which were chosen as the basic requirements for the final assessment instrument.

**Table 1:** Basic requirements for the final assessment instrument.

Face validity	Displaying full spectrum of mobilisation Inclusion of positioning and transfer abilities
Logical validity	Focus on the independence of functioning Item definition and item-to-item differentiation by established classification categories (based on ICF categories and subcategories of the ICF chapter d4, mobility [1])
Applicability and user friendliness	Simple clinical applicability Hierarchical composition Interprofessional applicability Assessment integration in regular therapy procedures Time efficiency of scoring

With the aim of better clinical applicability, the intra-item scaling of the pilot instrument was abolished as a condition for a hierarchical item sequence. The general item selection was maintained; however, the item *walking* needed further differentiation to avoid a bunching of scores in this item. The item sequence was arranged in hierarchy according to levels of mobility by logical judgement. The goal was to represent the steps of the most realistic sequence of mobility re-gain during a mobilisation and rehabilitation process. Consecutively the sequence of every two items in a row was verified on the basis of the previously acquired mobilisation profiles. Finally, a short manual was compiled, and a logical revision of the score with the acronym CHARMI was performed.

### Validation Phase

Before testing the psychometric properties of the CHARMI, content validity was re-evaluated by a panel of subject matter experts. Eight experts (four physicians and four physiotherapists, each with at least 3 years of experience in ACR), who were not involved in the development process of the instrument, answered a dichotomous question about the concordance of the instrument and the measured construct. Content validity was quantified using Lawshe’s method [38]. The content validity ratio (CVR) is the transformation of a proportional level of agreement depending on how many experts within a panel rate an item as being essential. CVR values range between −1 (perfect disagreement) and +1 (perfect agreement) and are put into relation with a reference value CVR_critical_, which is 0.75 in a panel with eight experts [38,39].

For further psychometric testing, CHARMI was applied on a sample of consecutive immobile patients of the same ACR programme for 12 months. All patients, who were immobile at admission (i.e. who were not able to stand or walk) and completed their individual ACR plan, were included (n = 87). Pre- and post-rehabilitation assessments using the CHARMI and Barthel Index were conducted. Sample characteristics and normative data were analysed.

Construct validity was calculated using a correlation of the CHARMI and Barthel Index. In comparison, the correlation of the CHARMI and the score of the three isolated mobility items of the Barthel Index (*transfers bed to chair and back*, *mobility on level surfaces* and *ability to climb stairs*) was calculated. Although the Barthel Index as a whole includes ADL and body functions which are not related to mobility, the correlation with the isolated Barthel Index mobility items indicates convergent construct validity. Confidence intervals of the correlation coefficients were calculated using Fisher’s transformation.

The assessments at admission as well as the re-assessments using the CHARMI at patients’ discharge included floor and ceiling effect calculations. Floor effects appear when there is a bunching of scores at the lowest index values (relevant at admission), whereas ceiling effects are measured by the percentage of the highest possible value (relevant at discharge). A percentage up to 20% is considered acceptable; more than 20% is poor [40].

Responsiveness to change was determined by interferential statistics using the paired sample Wilcoxon test. The distribution-based parameter of Cohen’s *d* was calculated to complement the hypothesis testing with an effect size as an indicator for the measure’s clinical relevance. In a simplified grading, ǀ*d*ǀ values over 0.8 represent a large effect size [41].

In a subgroup of 30 patients, the inter-rater reliability between the rating of a physician and a physiotherapist was tested using Cohen’s kappa statistics. Kappa describes the concordance of two variables with a value between 0 and 1, where higher values represent a higher concordance [42]. The testing was conducted consecutively until the defined subgroup sample size was reached. Despite possible differences in the patients’ performance the testing was performed as a test-retest situation to reach optimal blinding of the assessors.

The ACR plan and the ACR length of stay are individually planned and adjusted to the patients’ functional status, including their mobility level. CHARMI scores at admission were tested for their prognostic validity with regard to the length of stay in ACR. This was determined by Spearman’s rank correlation coefficient, where a positive or negative coefficient between +1 and -1, unequal to 0, quantifies the predictive value.

For reasons of patient safety, all assessments were performed in regular physiotherapy units, except for assessments performed by the physician during the inter-rater reliability tests. All testers were intensively trained in the application of the CHARMI and were experienced in mobilisation and ACR. The staff involved in the instrument’s development and validation statistics were not involved in the assessment procedures.

The German language version was used for the validity tests. An English-language version was established using a structured translation, re-translation and comparison process [43].

The study is registered at the German Clinical Trials Register (Registration number DRKS00010046). The institution’s ethics committee approved the study (vote number EA1/234/12). The participants provided general written consent to the use of their (pseudonymised) data for scientific reasons together with their respective medical treatment contract. The medical data used in this study was data of the clinical routine, obtained and used in the routine treatment processes, therefore no additional written consent was needed to be obtained for its analysis. The ethics committee approved this procedure according to the data protection law of Berlin (Berliner Datenschutzgesetz), provided that personal data is pseudonymised, pseudonymised data is not provided to a third party, and the project is limited to the concerned hospital. Thus, restrictions apply concerning data availability. Data are available from the Charité Universitätsmedizin Berlin Institutional Ethics Committee, Charitéplatz 1, 10117 Berlin, Germany, for researchers who meet the committee's criteria for access to the data (contact via the corresponding author; email to max.liebl@charite.de).

The data were analysed using SPSS Statistics version 22 (IBM, Chicago, IL, USA) and MS Office Excel 2010 (Seattle, IL, USA).

## Results

The revision of the pilot instrument by means of the above mentioned criteria resulted in a scale from complete immobility to full mobility. Each item was given a rank, beginning from 0 (represents complete immobility).

Item-to-item differentiation was elaborated using the categories and subcategories of the chapter d4 (mobility) in the ICF [1]. Due to their therapeutic relevance in the early course of mobilisation, the mobility items regarding transfers were divided into the following: *transfers in bed*, defined as rolling from the back to the side; active *transfer to edge of bed*; the ability to maintain a sitting body position (*sitting on edge of bed*); and *standing up*. The *transfer from bed to chair* was included as a separate item because patients with impaired functions of the lower extremities need this function as the basis of any independent wheelchair mobility. *Walking*, as the major locomotion ability, was differentiated into four separate items defined by walking distance based on the corresponding ICF subcategories. *Climbing stairs* was initially added as highest locomotive ability.

The item sequence was arranged according to hierarchy by logical judgement and then rearranged after double-checking the sequential arrangement of each two items according to the mobilisation patterns. Consequently, the items *sitting on edge of bed* and *transfer to edge of bed* were rearranged with the active transfer given the higher value, and the item *climbing stairs* was sorted in between *walking over 50 m* and *full mobility*.

The item *wheelchair mobility* could not logically be included in the hierarchical sequence. However, from a clinical and rehabilitation point of view, there was the need to consider it as a mobility skill of major importance. Therefore, we decided to operationalise *wheelchair mobility* outside of the sequential hierarchy. Independent wheelchair mobility is optionally encoded with an additional “W” after the digit of the assessment score.

The exemplary sequential conduct of the assessment was as follows. The patient starts in the lying position and is requested to turn from the back to the side (1 point) and then to sit up to the edge of the bed. If this is not possible, assistance will be given to sit up, and the patient is requested to sit at the edge of the bed freely for 30 seconds (2 points). Sitting up and sitting freely for 30 seconds without assistance receives 3 points. As a next step, the patient is asked to transfer to a chair or wheelchair (4 points) and then to stand up and keep the upright standing position for 30 seconds (5 points). Then locomotion abilities are the next sequential tasks. The patient is requested to start walking, and the walking distance is measured in three ICF-based distance sections (6 points: within the room, under 10 m; 7 points: inside the home or within the ward, 10–50 m; 8 points: outside the home or the ward, over 50 m). Nine points are achieved by walking one flight of stairs, and 10 points are given for full mobility with walking long distances over 1 km.

The use of assisting appliances (e.g. sliding boards for transfer, walking aids, rollators) is permitted in the assessment setting. However, the autonomous accomplishment of the mobility tasks is crucial. Any help or assistance involving other persons counts as failed task. The highest continuously achieved value determines the achieved score.

A patient who is capable of walking with crutches independently, without being able to transfer into a standing position in the absence of assistance, cannot receive more than 4 points (transfer from bed to chair).

A logical review of the instrument with regard to the a priori defined criteria and inter-item discrimination, with the use of the ICF codes, was performed, and a short manual was written (Tables 2 and 3).

**Table 2:** Differentiation of CHARMI items by ICF subcategories.

Score	Item	ICF Group	Categories	Subcategories
0	Complete immobility	-	-	-
1	Transfers in bed	Changing and maintaining body position (d410-d429)	d420 Transferring oneself	d4201 Transferring oneself while lying
2	Sitting on edge of bed	Changing and maintaining body position (d410-d429)	d415 Maintaining a body position	d4153 Maintaining a sitting position
3	Transfer to edge of bed	Changing and maintaining body position (d410-d429)	d410 Changing basic body position d415 Maintaining a body position	d4103 Sitting d4153 Maintaining a sitting position
4	Transfer from bed to chair	Changing and maintaining body position (d410-d429)	d420 Transferring oneself	d4200 Transferring oneself while sitting
5	Standing up	Changing and maintaining body position (d410-d429)	d410 Changing basic body position d415 Maintaining a body position	d4104 Standing d4154 Maintaining a standing position
6	Walking up to 10 m	Walking and moving (d450-d469)	d450 Walking^a^	d4500 Walking short distances
7	Walking 10 to 50 m	Walking and moving (d450-d469)	d450 Walking d460 Moving around in different locations^a^	d4500 Walking short distances d4600 Moving around within the home
8	Walking over 50 m	Walking and moving (d450-d469)	d450 Walking d460 Moving around in different locations^a^	d4500 Walking short distances d4602 Moving around outside the home and other buildings
9	Climbing stairs	Walking and moving (d450-d469)	d455 Moving around^a^	d4551 Climbing
10	Full mobility	Walking and moving (d450-d469)	d450 Walking^a^	d4501 Walking long distances
+W	Wheelchair mobility	Walking and moving (d450-d469)	d465 Moving around using equipment	-

All displayed groups, categories and subcategories fall into chapter d4 (mobility) in the component d (activities and participation) of the ICF (International Classification of Functioning, Disability and Health).

^a^When using mobilisation aids, d465 (Moving around using equipment) also applies.

**Table 3:** Charité Mobility Index [43].

**CHARMI Short Manual** Count the best mobility item that can be performed without assistance. Aids may be used. Count wheelchair mobility separately (e.g. 4+W).		
0	Complete immobility	
1	Transfers in bed	turning from back to side
2	Sitting on edge of bed	sit ≥ 30 s, transfer may need assistance
3	Transfer to edge of bed	transfer into sitting position
4	Transfer from bed to chair	
5	Standing up	with standing for ≥ 30 s
6	Walking up to 10 m	e.g. within a room
7	Walking 10 to 50 m	e.g. within a ward or inside the home
8	Walking over 50 m	e.g. outside a ward or the home
9	Climbing stairs	≥ 1 flight of stairs
10	Full mobility	≥ 1 km
+W	Wheelchair mobility	

### Validation Phase

Table 4 shows the demographic data of the sample and the normative data of the ACR sample for CHARMI.

**Table 4:** Sample characteristics and normative data of ACR sample for CHARMI.

**Demographic data**^a^	**n = 87**
Female sex	47 (54%)
Age (years)	59 ± 16.3 [13–88]
Body mass index	27.1 ± 7.1 [15.2–50.9]
Hospital length of stay	60.5; 78.8 ± 59.1 [20–313]
ACR length of stay	29; 36.6 ± 28.8 [8–195]
Intensive care treatment before ACR (%)	27.6
**Normative data of ACR sample**^b^	**n = 87**
CHARMI at admission to ACR	3 [1;4]
CHARMI at dismissal	6 [5;8]
Barthel Index at admission to ACR	45 [30;55]
Barthel Index at dismissal	72.5 [60;75]
**Sample characteristics by transferring discipline**	**n (%)**
Orthopaedic surgery	25 (28.7%)
Traumatology	21 (24.1%)
Vascular Surgery	12 (13.8%)
Oncology	7 (8.0%)
Neurology	7 (8.0%)
Rheumatology	6 (6.9%)
Others	9 (10.3%)

^a^Median (where appropriate); average ± standard deviation [minimum—maximum]. ACR: acute care rehabilitation.

^b^Median; interquartile range.

Content validity was confirmed by independent experts in the field of ACR. The CVR was calculated with CVR = 1 and therefore lies above the critical value of 0.75, indicating concordance of the instrument and the measured construct. The convergent construct validity of the CHARMI with the three mobility-related items of the Barthel Index (*transfers bed to chair and back*, *mobility on level surfaces*, *stairs*) were confirmed as excellent (r = 0.93). The calculated correlation with the whole Barthel Index, which predominantly assesses non-mobility ADL functions, was considerably lower (r = 0.63).

Responsiveness to change was determined by comparison of the CHARMI values before and after ACR. The median CHARMI score at admission was 3 (interquartile range 1;4) and at dismissal 6 [5;8]. Seventy-eight of the 87 patients increased their score, nine stayed on the same level, and no score decreased (78 positive ranks, 0 negative ranks). Interferential statistics proved a highly significant change with a very large effect size (ǀ*d*ǀ = 1.7; p < 0.001).

The inter-rater reliability between one physician and one physiotherapist was tested in a subgroup (n = 30). In 8 of the 11 single items, there was an exact agreement between the two raters. In three items (*transfer to edge of bed*, *walking up to 10 m*, *walking 10 to 50 m*), there were score differences. The maximum difference was one score point. The overall inter-rater reliability was excellent (κ = 0.88).

Floor and ceiling effects occurred during admission in 20% of cases. This is interpreted as just adequate. With a percentage of 3%, the ceiling effects at discharge were not relevant.

The analysis showed a significant negative correlation between the CHARMI admission scores and the ACR length of stay (r = -0.53; p < 0.01). Hence, the CHARMI score can be interpreted as a prognostic parameter for the length of a patient’s ACR stay. Table 5 shows the major results.

**Table 5:** Major results: Psychometric properties of the CHARMI.

Content validity	CVR = 1
Convergent construct validity with Barthel Index mobility items	r = 0.93
(Discriminant) construct validity with whole Barthel Index	r = 0.63
Responsiveness to change	ǀ*d*ǀ = 1.7; p < 0.001
Inter-rater reliability (n = 30)	κ = 0.88
Floor, ceiling effects (admission)	20%, 0%
Floor, ceiling effects (discharge)	0%, 3%
Predictive value of CHARMI at admission for ACR length of stay	r = -0.53; p < 0.01

ACR: acute care rehabilitation.

The forward-translation of the German version into the English version was conducted by an independent health professional. Another person, also not part of the authoring team, conducted a back-translation. According to the simplicity of the items, there was a complete agreement of both the original and back-translated version.

## Discussion

### Relevance of the Study

The CHARMI is an assessment instrument to display the full range of mobility in therapy-relevant steps. It is based on independence in mobility and thus has an ability-oriented rather than a disability-oriented approach. The CHARMI uses ICF criteria to differentiate levels of mobility.

The CHARMI is a valuable addition to regularly used assessment instruments measuring functioning and mobilisation. Up until today there has not been a practical mobility assessment tool for measuring mobility in acute care rehabilitation. The CHARMI fills this gap. Its comprehensive design and easy application give it practical relevance. Its practical usability combines with an excellent representation of the actual status of mobility of the patient. Any given score value corresponds with a mobility level, and the score alone will allow for an interpretation of the functioning in a given situation.

Changes of mobility levels and the course of mobilisation can sensitively be depicted. Together with the hierarchical scoring in therapy-relevant steps this allows for a valid monitoring of mobilisation. The CHARMI can be used by interprofessional teams, which makes the instrument well suitable in clinical settings. It can be used for goal setting and goal attainment scaling, which are increasingly important aspects of physiotherapeutic and other mobilisation treatments.

### Discussion of the Results

The general scope of clinical measurements can be divided into discrimination, prediction and evaluation. The former two are usually evaluated with the classical test quality criteria objectivity, reliability and validity [44]. However, assessment instruments designed for evaluation additionally have to determine changes of the measured quantity sensitively. Hence, responsiveness to change is a quality criterion of major importance for them. The CHARMI excellently fulfils these criteria in the current sample and is therefore very well suitable for the operationalisation of the mobility gain through the course of early rehabilitation interventions. It also is the basis for the presumption of the transferability to other samples.

Before using the instrument in samples with (known or expected) distinct floor or ceiling effects, there should precede a logical verification of its applicability. In the intensive care, for example, assessments that measure assisted mobilisation or even tolerance of passively reached mobility should be recommended. However, the CHARMI may be of use in intensive care unit patients as a descriptive measure or with regard to mobilisation goal setting and goal attainment scaling.

### Structure and Items of the Instrument

The measured construct is the independent functioning in mobility by definition and supported by the tested content validity. To that effect, and in favour of clinical usability, some aspects of mobility are not taken into account in the assessment. For example, these are the dependency on mobilisation aids or assistance. In particular situations, it may be relevant to assess the efforts in personnel and time or to discriminate non-professional and professional assistance or even the necessity of more than one assisting person for mobilisation. Mental functions and mobility restriction due to medical needs, such as full or partial weight bearing, are only considered in the CHARMI as a limitation of independent mobility functions. Those aspects are left unconsidered mainly to avoid further complexity. The advantage is that the assessment gives a global rating of the everyday mobility range a patient can actually reach, whereas many other assessment instruments test detailed or partial mobility aspects without regarding the reality of everyday functioning. Despite the use of ICF items for the single ranks, ICF Qualifier scales were not used for scoring to maintain the instrument’s easy applicability. The same applies for any additional coding of environmental factors like barriers and facilitators or even performance- or capacity-encoding qualifiers.

The single items of the CHARMI correspond to categories and subcategories of the chapter d4 (mobility) of the ICF. For reasons of better responsiveness to change and to achieve better clinical usability, we decided to display the order of therapeutic mobilisation rather than the sequence of tasks a healthy person would perform while standing up. Between the items *sitting on edge of bed* and *transfer to edge of bed*, the transfer reached a higher value. A potential physiological explanation could be that an active contraction of the core muscles is needed to sit up, whereas sitting itself can be performed with less muscle strength. Neuronal and proprioceptive functions also influence the degree of trunk stability. The differentiation of both items is therefore therapeutically relevant.

The differentiation of “walking” is classified into three items, each with a specific distance range, defining the appropriate assignment based on the ICF subcategories d4500 (walking up to 10 m/short distances), d4600 (walking from 10 to 50 m/within the home) and d4602 (walking more than 50 m/outside the home).

The full score (10) was defined as the equivalent to the ICF subcategory d4501 (walking long distances). Accordingly, the required walking distance for full mobility would be more than 1 km. Strictly speaking, it represents a fourth “walking” item, and one could argue that it requires less functional ability to ambulate a kilometre than to climb stairs. Yet, in this sample, the mobilisation profiles clearly show that climbing stairs is being independently managed by the majority of patients before reaching the capacity for walking long distances.

The time needed for the assessment, when individually performed, may vary greatly and is dependent on the patient’s level of mobility. That may imply time spans of up to 15 minutes in some cases. However, the average testing time in the acute rehabilitation unit is less than 5 minutes, according to the authors’ experience. Assessment requires the availability of necessary technical appliances, such as walking aids regularly used by the patients and access to facilities for measuring the walking distance and the possibility of climbing stairs. For testing the transfer tasks, a bed or examination table and a chair are required. The assessment can be performed as clinical observation integrated into the regular physiotherapy units or during nursing interventions, thus avoiding loss of time.

### Limitations

Usually, assessment instruments are tested and validated with samples that are specified by a group of similar diagnoses. In this study, the sample was deliberately not selected according to the patients’ ICD (International Classification of Diseases)-based diagnoses. The focus was laid instead on the homogeneity of their functional loss in mobility. An acute rehabilitation programme with a mixed sample of patients was chosen because a wide range of mobility limitations was sought. On the other hand, the ACR unit is the appropriate setting for the longitudinal exploration of an instrument that assesses mobility. In future efforts, we aim to validate the CHARMI in different patient groups according to their diagnoses, irrespective of their functional status.

The authors aimed with utmost effort to separate the staff involved in the development from the persons performing the testing. Yet, this has to be taken into account as a potential bias, as well as the limitation to one location and one sample.

The floor effects at admission can formally be rated as adequate, but with 20% of all admission scores they are in fact very close to being rated as poor. Due to the characteristics of the sample at an acute rehabilitation programme, in which patients are transferred just because they are immobile, this can be regarded as logical. Otherwise, it can also indicate the need for a further differentiation of the item “complete immobility”. However, this would possibly impair the focus on independence, as discussed above concerning the use of CHARMI in the intensive care setting.

Due to the ordinal scale and a missing intra-item differentiation (e.g. with Likert scales), the CHARMI may be inferior to instruments with a (pseudo-)interval scale, such as the DEMMI, for scientific use, especially if statistical analyses require higher scale of measurement. However, in the clinical setting, where there is a need to assess the overall function and longitudinal mobilisation developments, the CHARMI may be more useful as it covers the therapeutic steps of mobilisation and has a high responsiveness to change.

### Current Use of the Instrument

The CHARMI is currently being introduced in three campuses within the Charité University Hospital in Berlin, Germany, as a standard assessment of mobility, as well as for goal setting and goal attainment scaling in physiotherapy. More than 160 physiotherapists apply the instrument in more than 40,000 patients per year on a computer-based documentation of the assessments and goals. The authors expect to be able to present more validation data in different samples and mobility-related studies in the future.

## Conclusion

The CHARMI is a valuable clinical instrument to assess the level of mobility when focussing on independent functioning. It displays a wide spectrum of mobility in therapeutically relevant steps and combines the advantage of clinical applicability with high test quality criteria.
